# HER2 and HER3 cooperatively regulate cancer cell growth and determine sensitivity to the novel investigational EGFR/HER2 kinase inhibitor TAK-285

**DOI:** 10.18632/oncoscience.23

**Published:** 2014-03-24

**Authors:** Shinji Takagi, Hiroshi Banno, Akira Hayashi, Toshiya Tamura, Tomoyasu Ishikawa, Yoshikazu Ohta

**Affiliations:** ^1^ Oncology Drug Discovery Unit, Pharmaceutical Research Division, Takeda Pharmaceutical Company Ltd., Fujisawa, Kanagawa, Japan

**Keywords:** HER2, HER3, EGFR, kinase inhibitor, drug sensitivity, TAK-285

## Abstract

The human epidermal growth factor receptor (HER) family plays a major role in cancer cell proliferation. Overexpression of these receptors occurs in various cancers, including breast cancer, and correlates with shorter time to relapse and lower overall survival. We recently reported that TAK-285, an orally bioavailable small molecule inhibitor of HER kinases, is not a p-glycoprotein substrate and penetrates the blood-brain barrier, suggesting favorable activity for the treatment of brain metastases. To identify the determinants of sensitivity to TAK-285, we examined the relationship between the IC_50_ values of TAK-285 for cell growth inhibition and the expression of candidate genes that are involved in the HER family signaling pathway and trastuzumab resistance in a panel of human breast cancer cell lines, other types of cancer cells, and non-transformed cells *in vitro*. These analyses showed an inverse correlation between sensitivity to TAK-285 (IC_50_ values) and *HER2* or *HER3* expression. HER3 was highly phosphorylated in TAK-285-sensitive cells, where TAK-285 treatment reduced HER3 phosphorylation level. Because HER3 does not possess kinase activity and a selective inhibitor of HER2 but not of an epidermal growth factor receptor reduced the phospho-HER3 level, HER3 was suggested to be *trans*-phosphorylated by HER2. HER3 knockdown using small interfering RNA (siRNA) inhibited cancer cell growth in TAK-285-sensitive cells but not in TAK-285-insensitive cells. These results suggest that HER2 and HER3 mainly regulate cancer cell growth in TAK-285-sensitive cells and that phospho-HER3 could be used as a potential molecular marker to select patients most likely to respond to TAK-285.

## INTRODUCTION

HER (ErbB) family consists of epidermal growth factor receptor (EGFR; HER1, ErbB1), HER2 (ErbB2), HER3 (ErbB3), and HER4 (ErbB4). These receptors possess intrinsic tyrosine kinase activity within the intracellular domain except HER3. After binding of ligands such as epidermal growth factor (EGF) or heregulin to receptors, biological effects are exerted through homo-or heterodimerization [1, 2].

EGFR was identified as a receptor for EGF with high similarity to the v-erbB oncogene of avian erythroblastic leukemia virus [3]. EGFR is overexpressed in various tumors and correlates with patients' poor prognosis [4]. HER2 was originally identified as an activated protein in rat neuroblastoma, and it possesses homology to EGFR [5, 6]. Moreover, isolated cDNA has transforming activity [7]. HER2 is overexpressed in approximately 25% of breast cancer patients and is a poor prognostic marker [8]. HER2 is also overexpressed in various cancers such as ovarian, lung, and prostate cancers [9]. In clinical settings, EGFR kinase inhibitors such as gefitinib and erlotinib are used for advanced non-small cell lung cancer. The anti-HER2 antibody trastuzumab is used for the treatment of HER2-overexpressing breast cancer patients and the EGFR/ HER2 kinase inhibitor lapatinib is used for the treatment of HER2-overexpressing metastatic breast cancer patients who have progressed on trastuzumab therapy. Although HER3 does not possess intrinsic kinase activity, HER3 can form heterodimers upon ligand binding and activate the phosphatidylinositol 3-kinase (PI3K) pathway. HER3 is also believed to be an attractive drug target for cancer therapy [10].

Molecular targeted drugs have been tested in clinics and to identify patients likely to respond to the drugs is of importance for receiving clinical benefit from them. For example, Abl kinase inhibitors such as imatinib and nilotinib impede the proliferation of cells expressing the BCR-ABL fusion protein *in vitro* and have been proven to be beneficial on the population in clinical settings [11, 12]. Moreover, the clinical benefits of anti-HER2 therapies have been shown in patients with HER2 overexpression or *HER2* amplification, confirmed by immunohistochemistry and fluorescence *in situ* hybridization (FISH), respectively.

We recently reported that the novel investigational EGFR/HER2 kinase inhibitor TAK-285, which has anti-tumor activity and penetrates the rat blood-brain barrier, might be used for the treatment of HER2-overexpressing metastatic breast cancers [13-17]. We here searched for the determinants of sensitivity to TAK-285 and revealed high HER3 phosphorylation in TAK-285-sensitive cells. Subsequent pharmacological and siRNA experiments demonstrated that HER3 is mainly phosphorylated by HER2 and not by EGFR and that it plays an important role in the proliferation of TAK-285-sensitive cells. Therefore, phospho-HER3 could be used as a potential biomarker to select patients likely to respond to TAK-285.

## RESULTS

### HER3 or HER2 gene expression is inversely correlated to IC_50_ values of TAK-285

**Figure 1:**
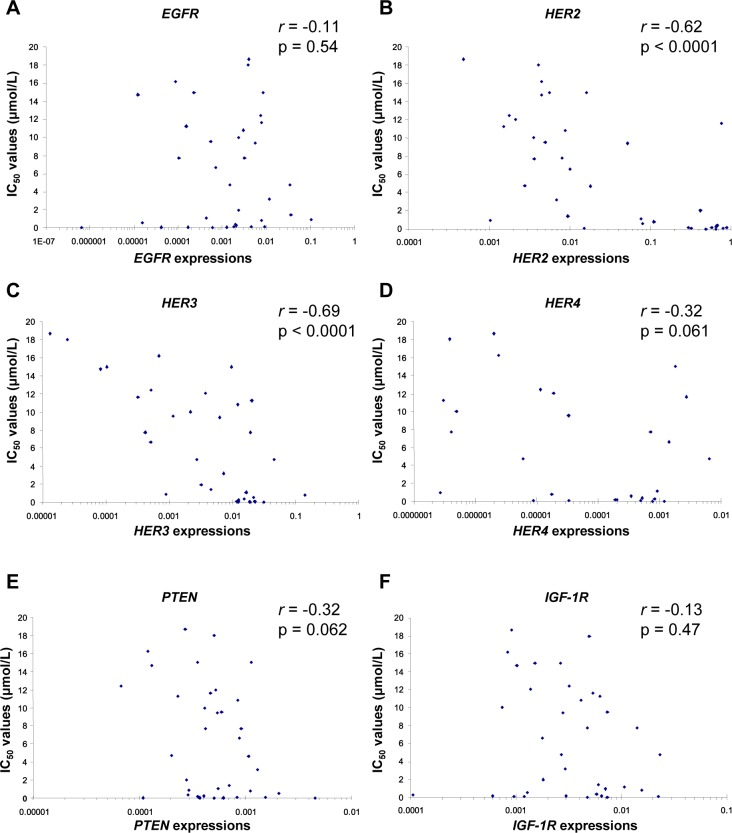
An inverse correlation between HER2 or HER3 gene expression and IC_50_ values of TAK-285 for cell growth inhibition Cell growth inhibition assays were performed using 35 human cell lines. Gene expression levels were determined by quantitative PCR and were normalized to GAPDH gene expression levels.

To identify the determinants of sensitivity to TAK-285, we examined the relationship between the IC_50_ values of TAK-285 for cell growth inhibition and EGFR, HER2, HER3, HER4, phosphatase and tensin homolog (PTEN), and IGF-1R gene expression in a panel of human breast cancer cell lines, other types of cancer cells, and non-transformed cells *in vitro*. These genes were selected because they reportedly regulate the HER family signaling pathway and trastuzumab resistance [18, 19]. TAK-285 dose-dependently inhibited the proliferation of all cell lines tested. The IC_50_ values of TAK-285 were determined in 35 cell lines and ranged widely (0.011~17 μmol/L), as described in Supplementary Table 1. Pearson's coefficient (*r*) indicated an inverse correlation between the IC_50_ values of TAK-285 and HER2 or HER3 gene expression (Figure 1).

### HER3 is highly phosphorylated in TAK-285-sensitive breast cancer cell lines

**Figure 2:**
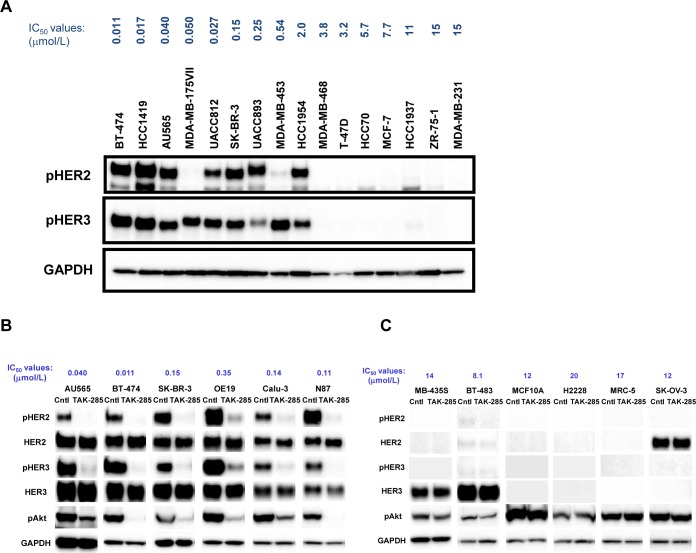
HER3 is highly phosphorylated in TAK-285-sensitive cells but not in TAK-285-insensitive cells A. Immunoblot analyses of 16 breast cancer cell lines using antibodies to phospho-HER2, phospho-HER3, and GAPDH. IC_50_ values of TAK-285 are presented in blue above the names of cell lines. B. TAK-285-sensitive cells were treated with dimethyl sulfoxide (DMSO) (control) or 1 μmol/L TAK-285 for 2 h. Phospho-HER2, phospho-HER3, and phospho-Akt levels were determined using immunoblot analyses. C. TAK-285-insensitive cell lines were used. The experimental procedure was the same as that in Figure 2B.

**Figure 3:**
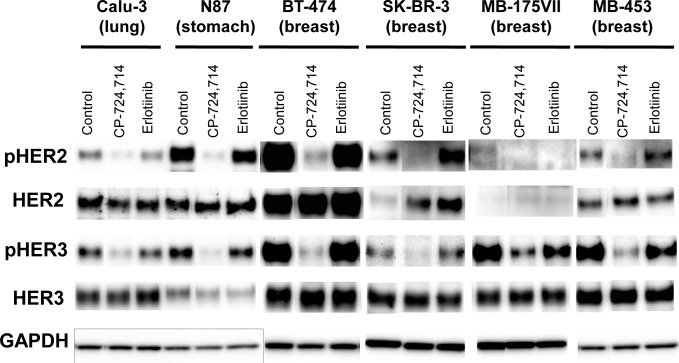
HER3 is *trans*-phosphorylated by HER2 but not by EGFR Calu-3, NCI-N87, BT-474, SK-BR-3, MDA-MB-175VII, and MDA-MB-453 cells were treated with DMSO (control), the selective HER2 kinase inhibitor CP-724,714, or the selective EGFR inhibitor erlotinib at 0.5 μmol/L for 2 h. Sufficient inhibition of EGFR phosphorylation by erlotinib was confirmed at this concentration (data not shown). Phospho-HER2 and phospho-HER3 levels were examined using immunoblot analyses. GAPDH was used as a loading control.

Immunoblot analyses showed coexpression of HER2 and HER3 in TAK-285-sensitive cell lines (Supplementary Figure 1). We assumed that HER2 and HER3 cooperatively regulate the proliferation of TAK-285-sensitive cancer cells. We determined the relationship between the IC_50_ values of TAK-285 and phospho-HER3 expression levels in 16 breast cancer cell lines. Phospho-HER3 was detected in TAK-285-sensitive cells (Figure 2A). This result shows that HER3 is activated in these cells. HER2 was not highly expressed in MDA-MB-175VII cells, but HER3 was highly phosphorylated (Figures 2A and 3). Wilson et al. recently showed that HER3 is activated by its ligand neuregulin-1 (NRG-1) in MDA-MB-175VII cells [20]. To investigate the effect of TAK-285 on the phospho-HER3 level, TAK-285-sensitive cell lines were treated with TAK-285 and immunoblot analysis was performed. TAK-285 treatment reduced the level of both phopho-HER2 and phospho-HER3 in sensitive cell lines (Figure 2B). HER3 possesses at least six PI3K binding sites and plays an important role in activating the Akt signaling pathway [21, 22]. Therefore, we examined phospho-Akt levels in cell lysates and found that the levels were clearly decreased after TAK-285 treatment of TAK-285-sensitive cell lines (Figure 2B). In A431 cells that overexpress wild-type EGFR, TAK-285 treatment led to reduced phospho-HER3 and phospho-Akt levels, despite the absence of phospho-HER2 or HER2 (Supplementary Figure 1). In contrast, phospho-HER2 and phospho-HER3 were not detected, and phospho-Akt was not decreased in TAK-285-insensitive cell lines after the treatment of TAK-285 (Figure 2C). These data indicate that phospho-HER3 could be a potential predictive marker for TAK-285 sensitivity.

It is known that HER3 does not possess intrinsic kinase activity and is *trans*-phosphorylated by EGFR or HER2 [23, 24]. To determine which members of the HER family phosphorylate HER3, we treated TAK-285-sensitive cells with the selective EGFR and HER2 kinase inhibitors erlotinib and CP-724,714, respectively [25, 26] and performed immunoblot analyses of phospho-HER2 and phospho-HER3. CP-724,714, but not erlotinib, reduced phospho-HER2 and phospho-HER3 in TAK-285-sensitive cells (Figure 3). In addition, erlotinib showed weaker anti-proliferative effects than CP-724,714 in the TAK-285-sensitive cell line HCC1419 (Supplementary Figure 1). These observations suggest that HER3 is phosphorylated by HER2 in most TAK-285-sensitive cells and that HER2:HER3 heterodimer plays important roles in the proliferation of these cells.

### HER3 positively regulates proliferation of TAK-285-sensitive cells

**Figure 4:**
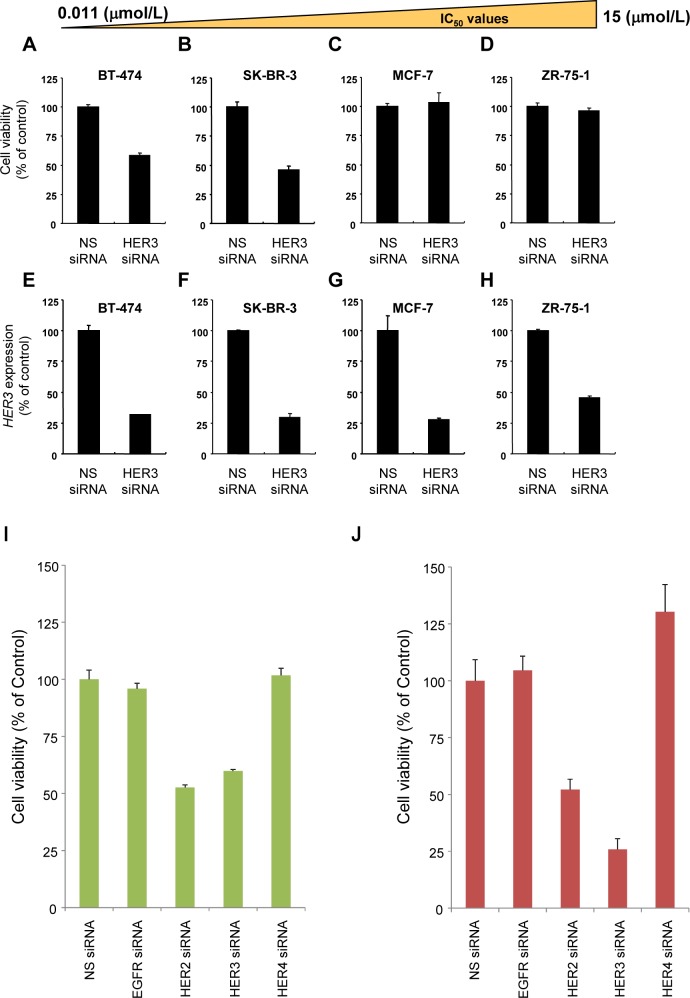
HER3 knockdown leads to growth inhibition in TAK-285-sensitive cells but not in TAK-285-insensitive cells Cells were transfected with 5 nmol/L NS or HER3 siRNA. Cell growth inhibition assays were performed (Upper panel, A-D), and HER3 gene expression was quantified relative to GAPDH gene expression (Lower panel, E-H). BT-474 (A, E) and SK-BR-3 (B, F) cells were used as TAK-285-sensitive cell lines. MCF-7 (C, G) and ZR-75-1 (D, H) cells were used as TAK-285-insensitive cell lines. BT-474 (I) and SK-BR-3 (J) cells were transfected with NS, EGFR, HER2, HER3, or HER4 siRNA. Growth inhibition assays were performed 5 days after transfection. Data are represented as the mean ± SD.

To investigate the involvement of HER3 in cancer cell proliferation, we examined the effects of HER3 knockdown in TAK-285-sensitive and -insensitive cells. The cells were transfected with nonsilencing siRNA (NS siRNA) or HER3 siRNA. Cell growth inhibition assays (Figure 4, Upper panel, A-D) were performed, and HER3 gene expression was quantified relative to GAPDH gene expression by quantitative PCR (Figure 4, Lower panel, E-H). HER3 protein knockdown was confirmed by immunoblot analysis (Supplementary Figure 2). BT-474 and SK-BR-3 cells were used as TAK-285-sensitive cell lines. MCF-7 and ZR-75-1 cells were used as TAK-285-insensitive cell lines. Although the level of HER3 mRNA expression was reduced after HER3 siRNA transfection in all cell lines tested, cell proliferation was inhibited only in TAK-285-sensitive cell lines, suggesting that HER3 positively regulates the growth of TAK-285-sensitive cells. The HER family consists of EGFR, HER2, HER3, and HER4. Therefore, we examined the effects of knockdown of individual HER family members on the proliferation of TAK-285-sensitive cells. The BT-474 and SK-BR-3 cells were transfected with NS or HER-specific siRNA and growth inhibition assays were performed. Specificity of each siRNA was confirmed by quantitative PCR analysis (Supplementary Figure 2). In these experiments, siRNA-mediated HER2 or HER3 knockdown, but not EGFR or HER4, inhibited the proliferation of TAK-285-sensitive cells (Figure 4I and J), indicating upregulation of HER2:HER3 signaling in TAK-285-sensitive cell lines. These data further indicate that phospho-HER3 could be used as a potential molecular marker for selecting patients likely to respond to TAK-285.

## DISCUSSION

Selection of patients likely to respond to molecular targeted therapeutics is of increasing clinical importance. For example, approximately 4% of non-small-cell lung cancers express the EML4-ALK fusion protein, providing a marker for responsiveness to the ALK kinase inhibitor crizotinib [27, 28]. Indeed, predictions of responses to molecular targeted drugs would increase the benefits for responsive individuals and the opportunities to protect them from unnecessary adverse effects.

The present data show that HER2 and HER3 are coexpressed and positively regulate the proliferation of TAK-285-sensitive cell lines. Moreover, HER3 was highly phosphorylated in TAK-285-sensitive cells and TAK-285 treatment reduced the phospho-HER3 level in these cells. Thus, we propose that phospho-HER3 could be used as a molecular biomarker to select patients likely to respond to TAK-285. Lee-Hoeflich et al. reported that the phospho-HER3 level is highly upregulated in *HER2*-amplified breast cancer patient tissues [29]. We speculated that HER2:HER3 heterodimer plays an important role in the maintenance of cell proliferation in not only cultured cell lines but also clinical settings. Although other HER family members might be involved in cell proliferation, the present pharmacological approach using a selective HER2 or EGFR inhibitor and RNA interference-mediated knockdown revealed that the HER2:HER3 heterodimer is a predominant regulator of the proliferation of TAK-285-sensitive cells. Engelman et al. and Buck et al. reported high HER3 expression and phosphorylation in EGFR kinase inhibitor-sensitive cancer cell lines [30, 31]. In agreement with previous study results, the present data suggest that HER3 is a common key regulator of the proliferation of EGFR-and HER2-dependent cancer cells.

We showed that MDA-MB-175VII cells with low HER2 expression are sensitive to TAK-285. Wilson et al. recently reported that this cell line overexpresses NRG-1 and demonstrated that a NRG-1-mediated autocrine loop activates HER3 via HER2 kinase [20]. Although we could not detect phospho-HER2 in MDA-MB-175VII cells by immunoblot analysis (Figure 2A), TAK-285 and CP-724,714 treatments reduced the phospho-HER3 level (Figure 3 and data not shown). These data suggest that weak HER2 activity might *trans*-phosphorylate HER3 and may be indispensable for the maintenance of the proliferation of MDA-MB-175VII cells. SK-OV-3 cells harbor both *HER2* amplification and *PIK3CA* (H1047R) mutation. In these cells, TAK-285 did not reduce the phospho-Akt level (Figure 2C), suggesting that the SK-OV-3 cells are resistant to TAK-285 because of *PIK3A* mutation-mediated activation of the Akt pathway or the lack of HER3 expression (Figure 2C). In this cell line, the Akt signaling pathway could be activated by *PIK3CA* mutation irrespective of *HER2* amplification. Loss of *PTEN* or *PIK3CA* mutation confers resistance to trastuzumab and lapatinib [32-34], although conflicting reports have been reported [35, 36].

Control of the HER3-Akt signaling pathway may be an important strategy for avoiding acquired resistance, as HER3 and Akt were reactivated after prolonged exposure to gefitinib [37]. HER3 and Akt were completely inhibited 6 h after TAK-285 treatment, whereas they were slightly reactivated 48 h after treatment (Supplementary Figure 3). In addition, TAK-285 treatment increased the HER3 protein level, similar to gefitinib treatment ([38] and Supplementary Figure 3). Further TAK-285 exposure may recover Akt activity via kinase-inactive HER3. Therefore, the combination of the Akt or PI3K kinase inhibitor with TAK-285 is a reasonable approach to prevent or delay drug resistance.

In this study, we analyzed the expression and phosphorylation of genes and proteins involved in HER signaling and identified a potential molecular marker for TAK-285 sensitivity. The present data indicate that patients likely to respond to TAK-285 might be identified prospectively on the basis of phospho-HER3 expression. Moreover, tumors with high phospho-HER3 levels appear to include TAK-285-sensitive subpopulations with and without *HER2*-amplification, such as MDA-MB-175VII cells with low HER2 expression.

## MATERIALS AND METHODS

### Cells and reagents

A2780, A375, A431, AU565, BT-474, BT-483, Calu-3, Cell System-Fb, H2228, HCC1419, HCC1937, HCC1954, HCC70, HCT116, HCC4006, HT-29, KYSE-30, K562, MCF-7, MCF-10A, MDA-MB-175VII, MDA-MB-231, MDA-MB-435s, MDA-MB-468, MDA-MB-361, MDA-MB-453 MES-SA, MES-SA/Dx-5, MRC-5, NCI-N87, NCI-H1781, OE19, OE21, OE33, OV-90, PC-3, SK-BR-3, SK-OV-3, T-47D, UACC812, UACC893, and ZR-75-1 cells were purchased from commercial sources and were maintained in media as prescribed by the suppliers. TAK-285 was synthesized at Takeda Pharmaceutical Company, Ltd. CP-724,714 was synthesized at Takeda Pharmaceutical Company, Ltd., according to published procedures [26, 39]. Erlotinib hydrochloride was extracted from Tarceva (Roche) at Takeda Pharmaceutical Company, Ltd.

### Growth inhibition assay

Cells were seeded into 96-well plates and treated with TAK-285 on the following day. Relative cell numbers were estimated by the sulforhodamine B staining method or the CellTiter-Glo assay (Promega). IC_50_ values for cell growth inhibition were calculated using SAS software (version 5.0).

### Quantitative PCR

Total RNA was extracted from each cell line using the RNeasy mini kit (Qiagen). Gene expression assays for EGFR, HER2, HER3, HER4, and glyceraldhyde-3-phosphate dehydrogenase (GAPDH) were purchased from Applied Biosystems. For data analysis, the Ct value was normalized to the Ct of GAPDH for each sample in order to obtain ΔCt and then the normalized ΔCt was calibrated to control samples to calculate ΔΔCt values.

### Immunoblot analysis

Cells were seeded into 24-well plates and treated with compounds for 2 h on the following day. Growth medium was removed and cells were lysed. Protein was resolved by SDS-PAGE and transferred to polyvinylidene fluoride membranes. The antibodies used in this study were specific for EGFR (#2232), HER2 (#2242), pHER2 (Tyr1248; #2247), pHER3 (Tyr1289; #4791), pAkt (Ser473; #9271, Cell Signaling Technology), HER3 (C-17, Santa Cruz Biotechnology), and GAPDH (ab9484-100, Abcam).

### Small interfering RNA (siRNA) transfection

Non-silencing (NS) control (Cat. No. 1022076), HER2 (Cat. No. SI02223571), and HER4 (Cat. No. SI00074193) siRNAs were purchased from Qiagen. EGFR siRNA (Cat. No. M-003114-01-05) and HER3 siRNA (Cat. No. sc-35327) were purchased from Dharmacon and Santa Cruz Biotechnology, respectively. Cells were transfected with 5 nmol/L siRNAs using LipofectAMINE 2000 (Invitrogen). Cell growth inhibition assays were performed, as described above, 3 or 5 days after transfection. Total RNA was extracted 2 days after transfection.

### SUPPLEMENTARY FIGURES AND TABLE


