# Supplementary material for: HER2 and HER3 cooperatively regulate cancer cell growth and determine sensitivity to the novel investigational EGFR/HER2 kinase inhibitor TAK-285
Source: Oncoscience. 2014 Mar 24;1(3):196–204. doi: 10.18632/oncoscience.23 (PMC4278294; doi:10.18632/oncoscience.23)
Supplement: Supplementary file 1 [file oncoscience-01-0196-s001.pdf]

# HER2 and HER3 cooperatively regulate cancer cell growth and determine sensitivity to the novel investigational EGFR/HER2 kinase inhibitor TAK-285 – Takagi et al

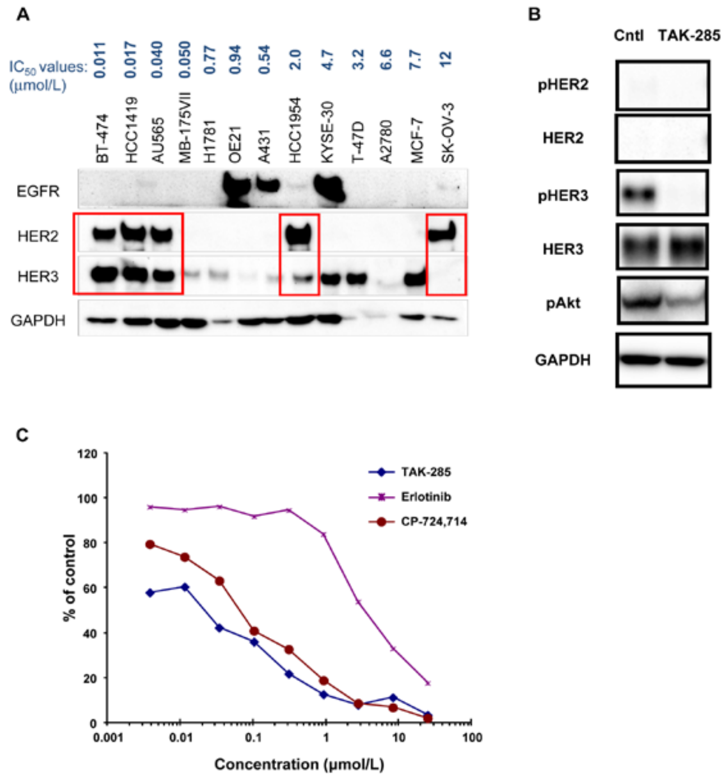


Supplementary Figure 1: HER2 and HER3 expression in cell lines. A. Coexpression of HER2 and HER3 proteins in TAK-285-sensitive cells. $IC_{50}$ values of TAK-285 are presented in blue above the names of cell lines. B. A431 cells were treated with 1 µmol/L TAK-285 for 2 h, the cells were lysed, and immunoblot analyses were performed. C. Cell growth inhibition assays were performed using HCC1419 cells, which express both HER2 and HER3.

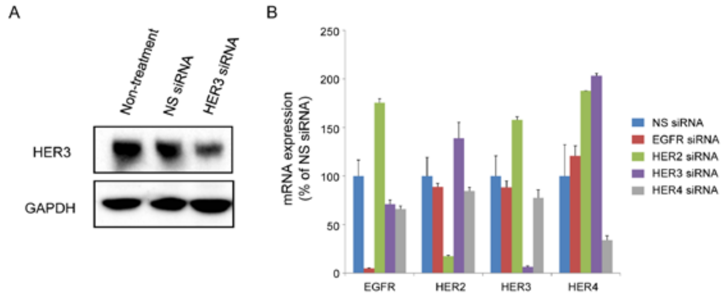


Supplementary Figure 2: Knockdown of the HER family using siRNAs. A. SK-BR-3 cells were transfected with siRNAs and lysed after 48 h incubation. Immunoblot analyses were performed using anti-HER3 antibody. GAPDH was used as a loading control. B. SK-BR-3 cells were transfected with siRNAs and total RNA was extracted 48 h after transfection. Quantitative PCR was performed.

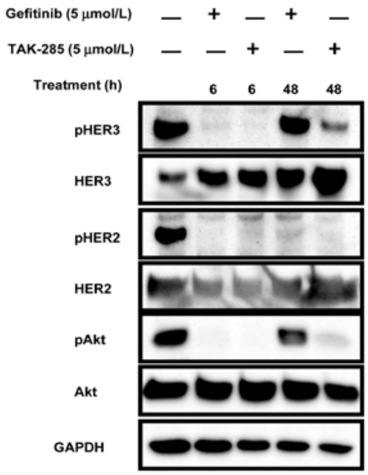


Supplementary Figure 3: HER3 reactivation in HER tyrosine kinase inhibitor-treated cells. SK-BR-3 cells were treated with 5 μmol/L gefitinib or TAK-285 for 6 or 48 h. Although gefitinib is best known as an EGFR kinase inhibitor, at this concentration it inhibited not only EGFR but also HER2 in cultured cells [37].

Supplementary Table 1: $IC_{50}$ values and HER family mRNA expression levels. $IC_{50}$ values of TAK-285 for cell growth inhibition and HER family, PTEN, and IGF1-R mRNA expression levels are presented.

| Tissue/Disease | Cell lines | $IC_{50}$ (μmol/L) | EGFR | HER2 | HER3 | HER4 | PTEN | IGF-1R |
|---|---|---|---|---|---|---|---|---|
| Breast | AU565 | 0.040 | 0.001253346 | 0.318640157 | 0.012516717 | 8.94802E-06 | 0.000109256 | 0.000956465 |
| Breast | BT-474 | 0.011 | 0.000618045 | 0.655196702 | 0.031467361 | 0.001202289 | 0.004549741 | 0.007340021 |
| Breast | MCF-7 | 7.7 | 0.000104805 | 0.007976657 | 0.019640834 | 0.000709948 | 0.000425074 | 0.014179987 |
| Breast | MDA-MB-175VII | 0.050 | 0.000167913 | 0.015092755 | 0.0119239 | 3.36275E-05 | 0.000372622 | 0.001185737 |
| Breast | MDA-MB-231 | 15 | 0.00837323 | 0.005640348 | 0.000104081 | 5.75E-07 | 0.001145347 | 0.002649618 |
| Breast | MDA-MB-361 | 1.1 | 0.000440064 | 0.077481731 | 0.016515907 | 0.000911165 | 0.000557014 | 0.01067219 |
| Breast | MDA-MB-453 | 0.54 | 1.53649E-05 | 0.079109787 | 0.021492841 | 0.000342882 | 0.002064488 | 0.001270842 |
| Breast | HCC1419 | 0.017 | 6.42411E-07 | 0.489710149 | 0.018971795 | 0.000766195 | 0.000509016 | 0.004775939 |
| Breast | HCC1954 | 2.0 | 0.00232267 | 0.420448208 | 0.003239529 | 3.74119E-07 | 0.000280444 | 0.001809742 |
| Breast | SK-BR-3 | 0.15 | 0.001835005 | 0.297301779 | 0.011841536 | 0.000190226 | 0.000357443 | 0.000596992 |
| Breast | T-47D | 3.2 | 0.001532391 | 0.018325546 | 0.046070913 | 0.006434305 | 0.001076079 | 0.023195681 |
| Breast | UACC-812 | 0.027 | 4.14003E-05 | 0.795536484 | 0.023035457 | 0.000491678 | 0.000618045 | 0.022561394 |
| Breast | UACC-893 | 0.25 | 0.002107868 | 0.655196702 | 0.01286861 | 0.000832651 | 0.000407758 | 0.000105534 |
| Breast | ZR-75-1 | 15 | 0.000232578 | 0.016064279 | 0.009752582 | 0.00182233 | 0.000352522 | 0.001511294 |
| CML | K562 | 12 | 1.61504E-06 | 0.002152158 | 0.003773189 | 1.87858E-05 | 0.000526967 | 0.001371528 |
| Colon | HCT116 | 10 | 0.002371474 | 0.003594483 | 0.002182201 | 5.00544E-07 | 0.00041345 | 0.000734984 |
| Colon | HT-29 | 11 | 0.003043612 | 0.008789519 | 0.012259127 | 8.18792E-07 | 0.00085606 | 0.004157696 |
| Epidermis | A-431 | 0.54 | 0.036397925 | 0.009485898 | 0.004613253 | 6.07758E-07 | 0.000709948 | 0.006003419 |
| Esophagus | KYSE-30 | 4.7 | 0.034196678 | 0.002781348 | 0.002724109 | 6.02754E-06 | 0.000201072 | 0.002686605 |
| Esophagus | OE19 | 0.35 | 0.001994164 | 0.673616788 | 0.015409886 | 0.000523327 | 0.000290334 | 0.00579892 |
| Esophagus | OE21 | 0.94 | 0.105112052 | 0.001046654 | 0.000911165 | 2.73871E-07 | 0.000296434 | 0.007089993 |
| Esophagus | OE33 | 9.4 | 0.005640348 | 0.052192995 | 0.006258358 | 4.06568E-07 | 0.00054555 | 0.002800694 |
| Lung | Calu-3 | 0.14 | 0.004613253 | 0.578344092 | 0.022405551 | 0.000201072 | 0.000838443 | 0.006479059 |
| Lung | H1781 | 0.77 | 0.0078125 | 0.110337875 | 0.140632311 | 1.77724E-05 | 0.001129578 | 0.015625 |
| Lung | HCC4006 | 3.2 | 0.011841536 | 0.006992383 | 0.007188966 | 5.29E-07 | 0.001297545 | 0.002919627 |
| Lung fibroblast | MRC-5 | 17 | 0.00390625 | 0.004128977 | 2.4788E-05 | 3.90006E-07 | 0.000512557 | 0.004978752 |
| Ovary | A2780 | 6.6 | 0.000709948 | 0.010237448 | 0.000516122 | 0.001410087 | 0.000880127 | 0.001784827 |
| Ovary | OV90 | 9.5 | 0.000549345 | 0.004978752 | 0.001153313 | 3.27079E-05 | 0.000596992 | 0.007391075 |
| Ovary | SKOV3 | 12 | 0.0078125 | 0.768437591 | 0.000324386 | 0.002762136 | 0.000471649 | 0.005336095 |
| Prostate | PC-3 | 12 | 0.007391075 | 0.001784827 | 0.000526967 | 1.1564E-05 | 6.77227E-05 | 0.003172861 |
| Skin | A375 | 11 | 0.000151332 | 0.001532391 | 0.020333466 | 2.99695E-07 | 0.000232578 | 0.006258358 |
| Skin | Cell | 7.7 | 0.003172861 | 0.0036194 | 0.0004192 | 4.15111E- | 0.0009175 | 0.0048091 |

| | | | | | | | | |
|---|---|---|---|---|---|---|---|---|
| | Systems-Fb | | | 85 | 21 | 07 | 03 | 58 |
| Stomach | NCI-N87 | 0.11 | 0.009226505 | 0.8827029 96 | 0.0188407 47 | 9.94173E-07 | 0.0015537 82 | 0.0030017 09 |
| Uterine | MES-SA | 15 | 1.23084E-05 | 0.0045497 41 | 8.39564E-05 | 5.59251E-07 | 0.0001317 42 | 0.0010180 33 |
| Uterine | MES-SA/Dx-5 | 16 | 8.93607E-05 | 0.0044871 03 | 0.0006953 37 | 2.41424E-06 | 0.0001212 27 | 0.0008269 |
